# Personalized 3D Printed Eye Gear for Microscopic Surgeons Amidst and beyond COVID-19

**DOI:** 10.3390/bioengineering10101129

**Published:** 2023-09-26

**Authors:** Ramandeep Singh, Rajdeep Singh, Ashish Suri

**Affiliations:** Neuro-Engineering Laboratory, Department of Neurosurgery, All India Institute of Medical Sciences, New Delhi 110029, India; ramanvirdis@gmail.com (R.S.); rajdeeps.me@gmail.com (R.S.)

**Keywords:** COVID-19, PPE, eye gear, field of view, 3D scanning, 3D printing

## Abstract

The COVID-19 pandemic underscored the need for enhanced protective measures for healthcare workers, particularly surgeons, who face a heightened risk of exposure to infectious aerosols. However, conventional eye protection equipment such as face shields, goggles, or glasses often leads to ergonomic discomfort and a reduced field of view (FOV), impeding surgeons’ ability to perform microsurgical procedures with precision and ease. To address these limitations, this study aimed to develop personalized 3D-printed eye gear for microscopic surgeons based on facial anthropometry data. 3D scanning was employed to obtain facial data from ten neurosurgery residents. Utilizing computer-aided designing, eye gears tailored to the unique facial features of each participant were developed. Finite element analysis-based contact simulation was used to assess the pressure exerted by the eye gear. Multi-material 3D printing was employed to fabricate the personalized eye gear. Participants, while donning the eye gear, engaged in simulation-based micro suturing tasks at various magnifications of the operating microscope, and marked the FOV range. They provided feedback scores (1–10) on the effectiveness of the eye gear through a Likert scale questionnaire (Q1-Q8). Finite element analysis demonstrated uniform strain distribution on the face, indicating that the edges of the customized eye gear fit exactly to the user’s face. The average scores for the questionnaire Q1 to Q8 ranged from 6.8 to 8.5, with an overall mean score of 7.6. This indicates that the developed eye gear was simple to use and did not cause any discomfort. Additionally, the average reduction in the FOV was only 10.93% across the different operating microscope magnifications. These findings highlight eye gear’s potential to alleviate discomfort and enhance precision in microscopic surgeries. Consequently, personalized 3D-printed eye gear offers a promising solution for providing surgeons with a safe environment while preserving the benefits of the operating microscope.

## 1. Introduction

The Severe Acute Respiratory Syndrome Coronavirus 2 (SARS-CoV-2) is the virus that causes the Coronavirus 2019 (COVID-19), which can cause respiratory tract infections and have serious health effects including death [1]. The outbreak started in Wuhan, Hubei, China, in December 2019, and was declared a pandemic by the World Health Organization (WHO) on 11 March 2020 [2]. As of May 2023, there have been 765,903,278 confirmed cases of COVID-19, including 6,927,378 deaths reported to the WHO, severely impacting the global healthcare system [3,4]. During a pandemic, the top priorities of a given healthcare system are the prevention of infection and the delivery of high-quality medical care to infected patients [5]. However, in such a situation, medical personnel on the frontlines, notably surgeons and paramedics, face the greatest risk of infection [6]. The nature of their work puts these healthcare professionals in direct contact with COVID-19 patients, increasing their vulnerability to infection with the virus. Surgeons, in particular, face an elevated risk due to various factors. These include their close proximity to patients, involvement in aerosol-generating procedures, prolonged exposure, a limited ability to maintain physical distancing, the handling of surgical instruments, and dealing with a high volume of patients [7].

Surgical specialties such as neurosurgery and ENT surgery require direct access to the nasal cavities and paranasal sinuses, making them more likely to produce infectious respiratory aerosols than coughing, sneezing, talking, or breathing [8,9]. Therefore, the high-risk nature of surgical operating rooms during such pandemics necessitates the deployment of additional protective measures. Routinely used personal protective equipment (PPE) for operating surgeons includes respirator masks, gowns with long sleeves, scrub caps, and gloves [10,11]. However, the Centre for Disease Control and Prevention (CDC) recommended additional eye protection PPE for surgeons. This recommendation was based on the observation that the presence of SARS-CoV-19 has been noted in conjunctival swabs taken from patients with COVID-19 [12,13]. Additionally, the expression of ACE2 and TMPRSS2 on the human ocular surface indicates potential vulnerability to SARS-CoV-2 infection through the conjunctiva [14]. Therefore, it is crucial that surgeons use eye protection PPE while performing surgery. However, the use of eye protection equipment such as face shields, goggles, or glasses has been found to cause ergonomic discomfort and a reduced field of view (FOV) while working with operating microscopes [15].

Operating microscopes are an integral part of modern microneurosurgical procedures [16]. They offer a high-definition vision of the surgical field, allowing surgeons to view intricate details with exceptional clarity. They include binocular vision that allows surgeons to view the surgical field with depth perception, providing a three-dimensional understanding of the anatomy. In addition to binocular vision, operating microscopes also incorporate an adjustable magnification system (2.83× to 17.71×) that allows for accommodating the surgeon’s individual visual needs [17]. These microscopes also provide a shared view between the operating surgeon and the assisting surgeon, ensuring effective collaboration and seamless communication during the procedure. During COVID, it was discovered that conventional eye protection methods increase the distance between the surgeon’s eyes and the operating microscope’s eyepiece lenses, thereby reducing the FOV [18]. Several studies have reported the widespread usage of exoscopes as surgical microscope alternatives to resolve incompatibility with eye protection PPE during COVID [19,20]. An exoscope is a type of surgical visualization system consisting of a camera attached to a long, slender arm that can be positioned above the patient’s head [21,22]. However, exoscopes have some disadvantages, such as loss of depth perception and difficult controls, leading to a steeper learning curve [23]. Thus, operating microscopes are preferable to exoscopes for neurosurgical procedures involving deep-seated tumors, as they provide surgeons with superior dexterity, performance, and operational comfort [24]. Consequently, an eye protection method that does not impede the microneurosurgical workflow is required.

COVID-19 caused disruptions in the supply chain and shortages of essential products, especially in the healthcare industry. In such a scenario, 3D printing emerged as an invaluable tool for the healthcare sector [25,26]. 3D printing, also known as additive manufacturing, is a technique that permits the rapid fabrication of three-dimensional objects through layer-by-layer addition of material. Fused deposition modeling (FMD), Selective laser sintering (SLS), and Material Jetting (PolyJet) are commonly used 3D printing techniques [27]. They allow fabrication in a range of materials including plastics, resins, composites, ceramics, and metals [28]. The versatility and ease with which 3D printing can produce customized objects have aided in the production of several healthcare components. During COVID, 3D-printing was used to produce PPE, medical devices, mask connectors, and other emergency resources [29]. Several types of 3D-printed visors for transparent face shields were developed [30]. Customized N-95 respirators that allows a consistent fit were also developed using 3D printing [31]. Additionally, 3D printing was used to produce nasopharyngeal swabs and sample collectors for COVID testing [32]. In addition, it was used to create CPAP mask connectors and other ventilator components [33]. The pandemic caused significant disruptions in the routine training of healthcare professionals, making it challenging to provide hands-on experience and practical training opportunities. Therefore, 3D printing was also used to create several training and visualization tools to train doctors [34]. Consequently, 3D printing emerged as an effective method to produce customized healthcare devices during COVID.

The present study aimed to develop 3D-printed surgeon-specific protective eye gear for microscopic surgeons. The design was based on the surgeon’s facial anthropometry data to ensure a perfect ergonomic fit. The eyewear was designed to protect the surgeon’s eyes during aerosol-producing operations, and to offer the largest feasible FOV range when using an operating microscope.

## 2. Materials and Methods

### 2.1. Materials

The fabrication of the eye gear necessitated the utilization of both soft and hard materials. Therefore, Digital ABS (Acrylonitrile Butadiene Styrene) and Agilus 30 materials (Stratasys, Rehovot, Israel) were employed in this study. Digital ABS is a photopolymer material specifically utilized in PolyJet technology. Its purpose is to emulate standard ABS plastic by combining RGD 515 and RGD 531 materials. According to Stratasys’s mechanical property datasheet, this material has excellent mechanical properties, including a tensile strength of 50–60 Mpa, a flexural strength of 65–75 Mpa, and a modulus of elasticity ranging from 2600–3000 Mpa. Thus, it is suitable for the development of products requiring high strength, temperature resistance, and superior finish. On the other hand, Agilus 30 serves as a rubberlike photopolymer material employed in Polyjet 3D printing. It is a flexible, durable, and versatile material with impressive mechanical properties, allowing for the creation of intricate and functional parts with fine details and high accuracy. Based on a Stratasys datasheet, it provides good tear resistance (4–7 kg/cm) and elongation at break (220–270%), making it ideal for applications requiring repeated flexing and bending and ensuring long-lasting durability. The material has a Shore A hardness of 30, which can be increased to 90 by combining it with Digital ABS [35,36].

### 2.2. 3D Scanning

**Figure 1:**
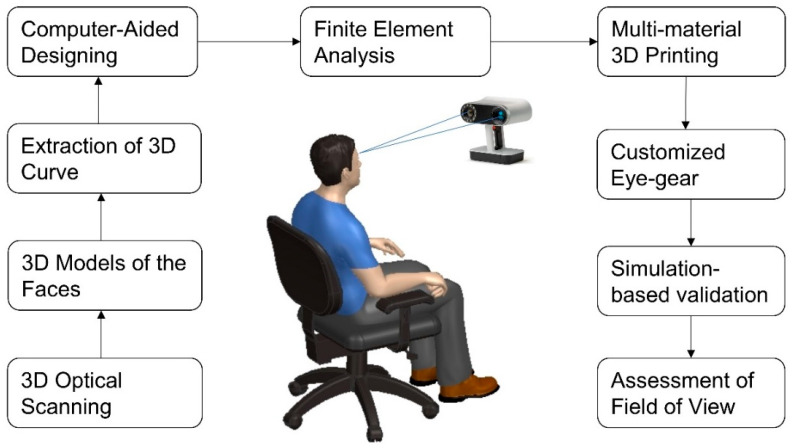
Methodology for the development and validation of personalized protective eye gear.

Ten senior neurosurgery residents were recruited for the present study after written consent (age: 30 ± 1.7 years). An Artec Leo 3D scanner was used to develop face models of these subjects. This scanner is a type of structured light scanner that captures detailed 3D images of objects. To capture the 3D images of the participants’ faces, the participants were asked to hold still while the scanner was used to capture multiple images from different angles. The scanner projected a pattern of light onto the participants’ faces, and the camera captured the pattern’s distortion as a result of the contours on the surface of the face. Artec Studio software was used to process scan data and create a detailed 3D model of each individual’s face. The complete methodology of development and validation of the customized eye gear is shown in Figure 1.

### 2.3. Computer-Aided Design

The customized eye gear for the study was developed using Geomagic Freeform (3D Systems Inc., Cary, NC, USA) software in conjunction with a haptic device. First, the scan data were loaded into the software, and a 3D curve was traced on the nose, temples, and forehead regions of the scanned surface. The points for the 3D curves were created using the stylus of the haptic device, which simulates the sensation of touching by providing force feedback. After extracting the 3D curve, it was refined by adjusting its shape and position using the curve’s smoothness and curvature operations. This step ensured that the 3D curve accurately represented the shape of the user’s face and eye socket, and served as a reference for modeling the customized eye gear for the participants.

**Figure 2:**
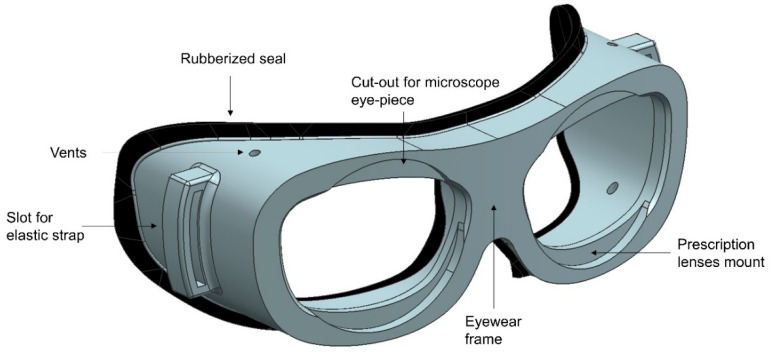
3D model of personalized eye gear with provision for the fixation of prescription glasses and slots for microscope eye-piece lens insertion.

The 3D model of the eye gear was designed with a 3 mm thick strip along the 3D curve. The strip was intended to provide a sealed environment that would prevent aerosols from entering the eyes. The eye gear frame model was designed to accommodate prescription glasses, ensuring that the user’s vision was not compromised. Additionally, two slots were made that could fit operating microscope eye-piece lenses, aiming to minimize the distance between the eyes of the surgeon and the microscope lenses. Two slots for securing the elastic strap were also designed on the sides of the eyewear to provide a secure fit. Finally, vents were incorporated into the design to reduce fogging, improving the user’s visibility and overall experience. The 3D model of the eye gear is depicted in Figure 2.

### 2.4. Finite Element Analysis

Finite element analysis (FEA) is a tool for validating engineering designs when physical measurements are difficult to obtain. It was difficult to manually determine the customized eyewear’s fitment and level of pressure exerted on the user’s face. Therefore, FEA was utilized to calculate the equivalent strain or pressure acting on the area underneath the eye gear supports. To perform the analysis, geometrical data from the 3D scanning of the face and the CAD model of the eye gear were used. The 3D models of the face and the eye gear were assembled, and a static structural FEA was conducted using ANSYS Mechanical software (Version: 2023 R1).

The skin on the face and forehead was assigned a thickness of 5 mm, and the entire posterior surface of the skin was assumed to have a fixed boundary. An anterior-posterior force of 4 N was applied to the participants’ faces, which would bear the primary loads due to the stretching of the eye gear’s strap. This allowed for the determination of the stress and deformation of the eye gear under load. The results of the FEA analysis were then used to optimize the design of the eye gear.

### 2.5. 3D Printing and Assembling

**Figure 3:**
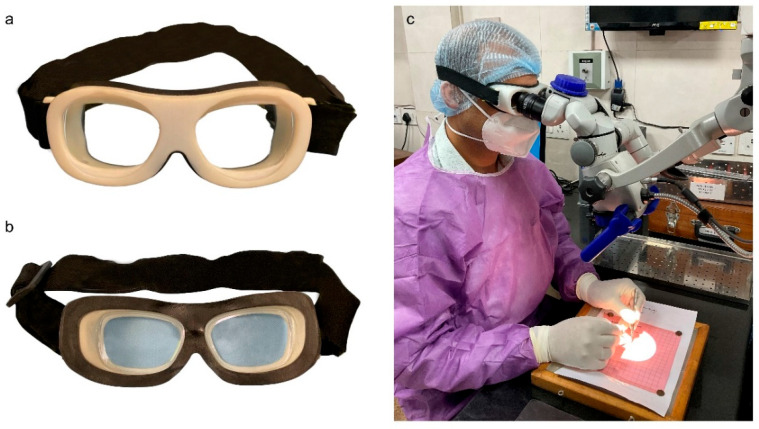
3D printed prototype of surgeon-specific protective eye gear: (**a**) front view, (**b**) back view, (**c**) the eye gear being used in conjunction with an operating microscope during simulation-based micro-suturing experiments.

The personalized eye gear prototypes were fabricated using a polyjet-based Objet260 Connex 3D printer (Stratasys, Rehovot, Israel), which is capable of fabricating multiple materials in a single print, resulting in an assembled prototype. The frame of the eye gear was fabricated using Digital ABS plastic material, and the rubberized seal was made using Agilus material, which had 60-shore hardness. The digital material mode of the GrabCad Print software, which allows printing with a 30-micron layer thickness, was selected. The total printing time required for the fabrication of one prototype was approximately 8.5 h. After the prototype was fabricated, it was manually fitted with the user’s prescription lenses and an elastic strap. For participants with normal vision, lenses without power were fitted. The assembled model of the surgeon-specific protective eye gear and the experimental setup are shown in Figure 3.

### 2.6. Subjective Validation

Simulation-based micro-suturing experiments were conducted to validate the developed eye gear. All participants performed a micro-suturing task at ten different magnifications (0.4 (2.83×), 0.5 (3.24×), 0.6 (4.25×), 0.7 (4.96×), 0.8 (5.67×), 1 (7.08×), 1.2 (8.49×), 1.6 (11.33×), 2.0 (14.17×) and 2.4 (17.71×)) using different sizes of monofilament nylon sutures and a Zeiss OPMI Vario Microscope (Carl Zeiss Inc., Oberkochen, Germany). The task included performing simple interrupted sutures and surgeon’s knot tying on rubber sheets. Post-experiment feedback was acquired using a Likert-type questionnaire, as depicted in Table 1. The questionnaire aimed to evaluate various aspects of the eye gear, including fit, comfort, visibility improvement, adjustability, the likelihood of continued usage, comparison to previous eye gear, reduction of eye fatigue, and enhancement of overall surgical performance. The questionnaire consisted of eight questions, each rated on a scale ranging from 1 to 10 (with 1 representing low satisfaction, discomfort, or improvement, and 10 representing high satisfaction, comfort, or improvement). The scores for each question were tabulated in a Microsoft Excel spreadsheet.

### 2.7. Field of View

FOV is a crucial aspect of any surgical procedure, and it refers to the largest area that a surgeon can view through the eyepiece of an operating microscope. However, the use of eye protection PPE affects the FOV. This is because the PPE increases the distance between the eyes and the eyepiece lenses of the operating microscope, thereby reducing the FOV [18]. To evaluate the impact of developed eye gear on the surgeon’s FOV, it was marked and compared at different magnifications of the operating microscope, both with and without eye gear. The markings were obtained on graph paper, and the area was calculated by counting the number of squares that fall within the marked region and multiplying by the area of each square. By doing so, we were able to determine the extent of the reduction in the FOV caused by the developed eye gear.

## 3. Results

### 3.1. Finite Element Analysis

**Figure 4:**
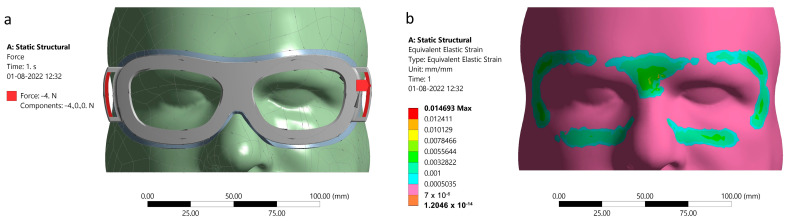
(**a**) Assembly of 3D scanning of the face and the CAD model of the eye gear with 4 N force applied at the strap slots, (**b**) FEA derived approximate equivalent strain distribution under the surgeon-specific protective eye gear.

The FEA results showed that the surgeon-specific protective eye gear effectively sealed the surgeon’s eyes. The force applied by the stretching of the eye gear strap was distributed evenly across the face, resulting in uniform strain on most of the underlying skin. However, slightly higher strain was observed in the areas near the supraorbital notches and zygomaxillary sutures, although this remained within an acceptable range. These findings indicate that the surgeon-specific protective eye gear can provide adequate protection to the surgeon’s eyes during surgical procedures. The assembly and approximate equivalent strain below the eye gear is shown in Figure 4.

### 3.2. Neurosurgeons Feedback

**Figure 5:**
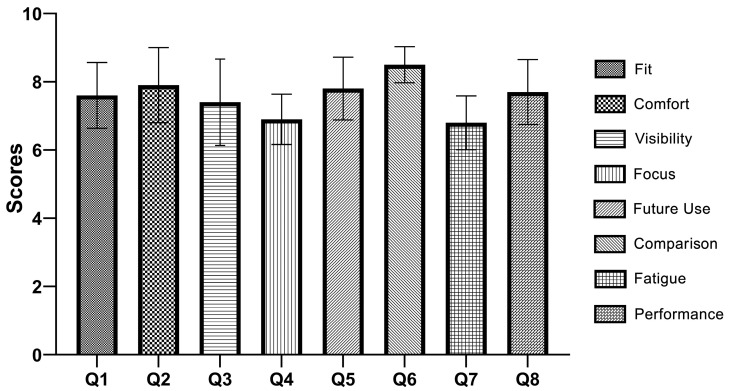
Participant feedback validation results on the personalized eye gear using the Likert scale questionnaire.

The results of the participant feedback show that the neurosurgeons were generally satisfied with the eye gear (Figure 5). The highest mean score was obtained for Q6 (average score = 8.5) (the comparison to other eye protection methods), which suggests that the developed eye gear was very effective as compared to other eye protection methods. The mean scores for questions 1, 2, 3, 5, and 8 were fairly close together, indicating that the neurosurgeons were generally positive about the fit, comfort, visibility improvement, likelihood of continued use, and overall improvement in surgical performance. However, the mean score for question 4 (the ease of adjusting focus) was lower than the others, suggesting that this aspect of the eye gear may need improvement. Similarly, the mean score for question 7 (the reduction of eye fatigue) was also lower than the others, suggesting that further research may be needed to improve this aspect of the eye gear.

### 3.3. Field of View

**Figure 6:**
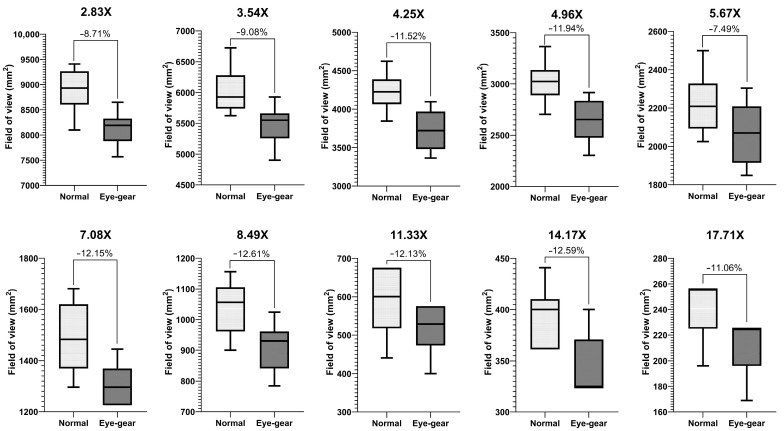
Comparison of the FOV at different magnifications of the operating microscope with and without the developed eye gear.

The analysis of the FOV area demonstrates the substantial advantage of the developed eye gear as compared to other conventional types of eye protection PPE [15]. The average FOV at 0.4 (2.83×) magnification of the operating microscope reduced from 8916 mm^2^ to 8139 mm^2^, with an average percentage reduction of 8.71%. Similarly, at higher magnifications, the average percentage reduction in FOV ranged from 12.58% at 2.0 (14.17×) magnification to 12.38% at 2.4 (17.71×) magnification. Figure 6 depicts the average percentage reduction in the FOV area when using the developed eye gear at various magnifications of the operating microscope. The data reveal an overall average reduction of 10.93% in the FOV area across different magnifications.

Overall, the results indicate that personalized 3D-printed eye gear offers a promising solution for surgeons, enabling them to perform microsurgical procedures with enhanced comfort and precision.

## 4. Discussion

The COVID-19 pandemic has had a profound impact on the healthcare sector and resulted in immense pressure on healthcare systems worldwide [37]. The global supply chain for medical equipment and supplies faced disruptions, highlighting the need for resilient and diversified supply chains [38]. There was a sudden demand for PPE, including masks, gloves, gowns, and face shields, during the pandemic. This highlighted the need for additional protective measures for healthcare workers, especially surgeons, who are at the highest risk of exposure to infectious aerosols [39]. In addition to other PPE, eye protection was essential, because the virus was found to be able to enter through the conjunctiva route [40]. However, the use of face shields, goggles, or glasses as eye protection equipment caused ergonomic discomfort and a reduced FOV while working with operating microscopes [41]. The decreased FOV makes it difficult to view the region of interest and manipulate instruments during surgery [15]. Surgeons require an unobstructed FOV to maintain their dexterity and operational comfort while performing microsurgical procedures [18]. This unobstructed view enables surgeons to precisely navigate around complex anatomical structures and manipulate instruments, thereby augmenting surgical performance and patient outcomes. Therefore, the standard eye protection apparatus was found to be incompatible with the operating microscopes [18].

Several studies have reported decreased FOV and ergonomic discomfort while wearing eye protection PPE. P. J. Clamp et al. reported the challenges faced by surgeons in performing mastoidectomy procedures while wearing PPE during the COVID-19 pandemic. They showed that the distance of the eye to the microscope lenses was inversely correlated with the visible area. The median area visible while wearing the full-face visor was only 4–16% [42]. A. Iyer et al. conducted an experimental study using a surgical microscope and a simulated surgical field to measure the FOV at different distances from the eyepiece and with different types of PPE. Their results showed that simple goggles reduced the FOV by up to 31.6%, large goggles by up to 75.7%, and face shields by up to 61.9% [15]. E. Celtikci et al. also showed that the use of eye protection equipment significantly reduced the FOV. They reported that the FOV of the naked eye was 9305.33 ± 406.1 mm^2^, with blast goggles was 2501.91 ± 176.5 mm^2^, and with face shields was 92.33 ± 6.4 mm^2^, whereas the FOV of the safety spectacles was 9267.45 ± 410.5 mm^2^. Therefore, safety spectacles were found to be most suitable for microscopic surgeries [18]. Consequently, there was a need to develop protective eye protection methods that can provide comfort, safety, and proper visualization during microscopic surgeries.

The present study aimed to develop personalized 3D-printed eye gear for microscopic surgeons during the COVID-19 pandemic. The use of 3D scanning allowed the design to be based on neurosurgery residents’ facial anthropometry data to ensure a perfect ergonomic fit for each participant. Static structural finite element analysis revealed that forces exerted by the eye gear were uniformly distributed across the user’s visage. Multiple-material 3D printing enabled the production of customized eyewear with a rubberized Agilus material seal and a rigid Digital ABS material frame. Depending on the user’s requirements, the 3D-printed eyewear was fitted with prescription or non-prescription lenses. The FOV was measured while the surgeons evaluated the developed eye gear by performing simulation-based micro-suturing experiments. When compared to other eye-protection methods documented in the literature, the personalized eye gear resulted in minimal reduction in the FOV while working on an operating microscope. This shows that the FOV was preserved to a great extent when using the personalized eye gear. Using a Likert-scale questionnaire, user feedback on the eye gear’s fit, comfort, visibility improvement, adjustability, the likelihood of continued use, comparison to previous eyewear, reduction of eye fatigue, and improvement of overall surgical performance was obtained. The feedback results indicate that the developed eye gear was easy to use and caused less fatigue to the participants. The participants reported no discomfort while wearing the eye gear, and reported that the developed eye-gear was superior as compared to other eye-protection methods. Therefore, surgeons recommended the use of personalized eye gear during aerosol-producing procedures.

The present study highlights the importance of preparedness for future outbreaks. Although the number of COVID-19 cases has significantly reduced, the study underscores the need for continued efforts in research and the development of protective measures to ensure safety of healthcare workers in the event of future pandemics. While the study has provided promising results, certain limitations should be acknowledged. Firstly, we did not conduct mechanical testing or biocompatibility assessments, which are important aspects to consider for ensuring the durability and safety of the eye gear. Additionally, our study only involved a small sample size of ten neurosurgery residents, which limits the generalizability of the findings. In future research, it would be beneficial to conduct larger-scale studies involving a diverse group of surgeons to validate the effectiveness and comfort of personalized eye gear. Nevertheless, the results of this study will serve as a valuable reference for health organizations and governments, providing practical solutions for eye protection PPE and reducing the risk of transmission of infectious diseases. Thus, this study is an important work for the future, as it will help to create a safer and healthier environment for all.

## 5. Conclusions

The present study demonstrates the potential of 3D scanning and printing techniques for developing personalized protective equipment that is both comfortable and functional. The developed personalized eye gear provides a promising approach to mitigate ergonomic discomfort while retaining the benefits of the operating microscope. It was able to provide a sealed environment to the eyes while minimizing the reduction in the surgeon’s FOV during surgical procedures. The findings of this study can significantly improve the safety and comfort of surgical personnel on the frontlines during pandemics. This technology can also be used to make eye gear for people with special needs, or for people working in extreme environments.

## 6. Patent

The patent application entitled “Three-Dimensional Printed Protective Eye Gear with Prescription Glasses” was filed with the Indian Patent Office on 29 April 2022. The application number is 202211025264.

## Figures and Tables

**Table 1:** Parameters for evaluation of surgeon-specific protective goggles.

S. No.	Questionnaire
1	How satisfied are you with the fit of your personalized eye gear?
2	How comfortable is your personalized eye gear while performing a surgical task?
3	How much does your personalized eye gear improve your visibility while working on the operating microscope?
4	How easy is it for you to adjust and make changes to your focus using your personalized eye gear?
5	How likely are you to continue using your personalized eye gear in future surgeries?
6	How does your personalized eye gear compare to other eye protection methods you have used in the past?
7	How much does your personalized eye gear reduce the fatigue on your eyes?
8	How much does your personalized eye gear improve your overall surgical performance?

## Data Availability

The data are not publicly available due to privacy restrictions.
